# Ketogenic diet enhances neurovascular function with altered gut microbiome in young healthy mice

**DOI:** 10.1038/s41598-018-25190-5

**Published:** 2018-04-27

**Authors:** David Ma, Amy C. Wang, Ishita Parikh, Stefan J. Green, Jared D. Hoffman, George Chlipala, M. Paul Murphy, Brent S. Sokola, Björn Bauer, Anika M. S. Hartz, Ai-Ling Lin

**Affiliations:** 1 Sanders-Brown Center on Aging, University of Kentucky, Lexington, KY 40536 USA; 2 Research Resources Center, University of Illinois at Chicago, Chicago, IL 60612 USA; 3 Deparment of Pharmacology and Nutritional Science, University of Kentucky, Lexington, KY 40536 USA; 4 Department of Molecular and Cellular Biochemistry, University of Kentucky, Lexington, KY 40536 USA; 5 Department of Pharmaceutical Sciences, University of Kentucky, Lexington, KY 40536 USA; 6 F. Joseph Halcomb III, M.D. Department of Biomedical Engineering, University of Kentucky, Lexington, KY 40536 USA

## Abstract

Neurovascular integrity, including cerebral blood flow (CBF) and blood-brain barrier (BBB) function, plays a major role in determining cognitive capability. Recent studies suggest that neurovascular integrity could be regulated by the gut microbiome. The purpose of the study was to identify if ketogenic diet (KD) intervention would alter gut microbiome and enhance neurovascular functions, and thus reduce risk for neurodegeneration in young healthy mice (12–14 weeks old). Here we show that with 16 weeks of KD, mice had significant increases in CBF and P-glycoprotein transports on BBB to facilitate clearance of amyloid-beta, a hallmark of Alzheimer’s disease (AD). These neurovascular enhancements were associated with reduced mechanistic target of rapamycin (mTOR) and increased endothelial nitric oxide synthase (eNOS) protein expressions. KD also increased the relative abundance of putatively beneficial gut microbiota (*Akkermansia muciniphila* and *Lactobacillus*), and reduced that of putatively pro-inflammatory taxa (*Desulfovibrio* and *Turicibacter*). We also observed that KD reduced blood glucose levels and body weight, and increased blood ketone levels, which might be associated with gut microbiome alteration. Our findings suggest that KD intervention started in the early stage may enhance brain vascular function, increase beneficial gut microbiota, improve metabolic profile, and reduce risk for AD.

## Introduction

Neurovascular functions play a critical role in determining cognitive capability and mental health^1^. Studies have shown that neurovascular risk is highly associated with accelerated decline in language ability, verbal memory, attention and visuospatial abilities^2,3^. Specifically, reduced cerebral blood flow (CBF) is linked to increased risk for anxiety, depression, and dementia^3–5^, while impaired blood-brain-barrier (BBB) function is associated with neuroinflammation, synaptic dysfunction, and psychiatric disorders^6,7^.

Emerging evidence suggests that gut microbiota play an important role in determining brain vascular integrity. Braniste *et al*. recently showed that BBB permeability is increased in germ-free mice due to lack of butyrate, a short chain fatty acid (SCFA) produced by microorganisms such as *Clostridium tyrobutyricum*^8^. Similarly, *Akkermansia muciniphila* can produce SCFAs such as acetate and propionate^9^, and the lack of *A. muciniphila* can alter the microbial ecology of the mucus layer^10^ and lead to damage of tight junctions of BBB and the gut^7,11^. BBB dysfunction further leads to reduced CBF and impaired clearance of amyloid-beta (Aβ) plaques, a hallmark of Alzheimer’s disease (AD)^1,6^. Interventions that maintain gut microbiome and neurovascular integrity may be thus crucial for impeding neurological disorders.

Ketogenic diet (KD), a high fat and low carbohydrate diet, has been an effective therapeutic for a wide range of neurological disorders^12^. Clinically, KD has been used to treat epilepsy^12,13^, Parkinson’s disease^14^, and autism^15^. Furthermore, significant evidence for KD as a therapeutic for a broader range of conditions can be found in preclinical studies where KD has been shown to protect brain function in Alzheimer’s disease^16^, traumatic brain injury^17^, and ischemic stroke^18^. A recent study showed that KD significantly increased regional CBF in a mouse model with ischemic stroke^19^. In another study with an autism mouse model, KD mitigated neurological symptoms potentially through changes in the gut microbiome^20^. Collectively, KD may be protective against various neurological disorders, possibly through the restoration of neurovascular function and by maintaining healthy gut microbiome.

While KD has beneficial effects in disease systems, it is unclear if similar impacts persist in healthy conditions. Therefore, the goal of the study was to identify whether administration of KD to young healthy mice would also benefit their neurovascular functions and gut microbiome composition, and whether these changes could contribute to lowering risk for AD, the most common form of dementia. We were also interested in identifying potential signaling pathways in association with vascular changes induced by KD. We hypothesized that KD would increase neuroprotection for young healthy mice, and reduce their risk for neurodegeneration, by enhancing their neurovascular functions and increasing the abundance of beneficial gut microbiota.

## Results

### Ketogenic diet enhances neurovascular functions

**Figure 1:**
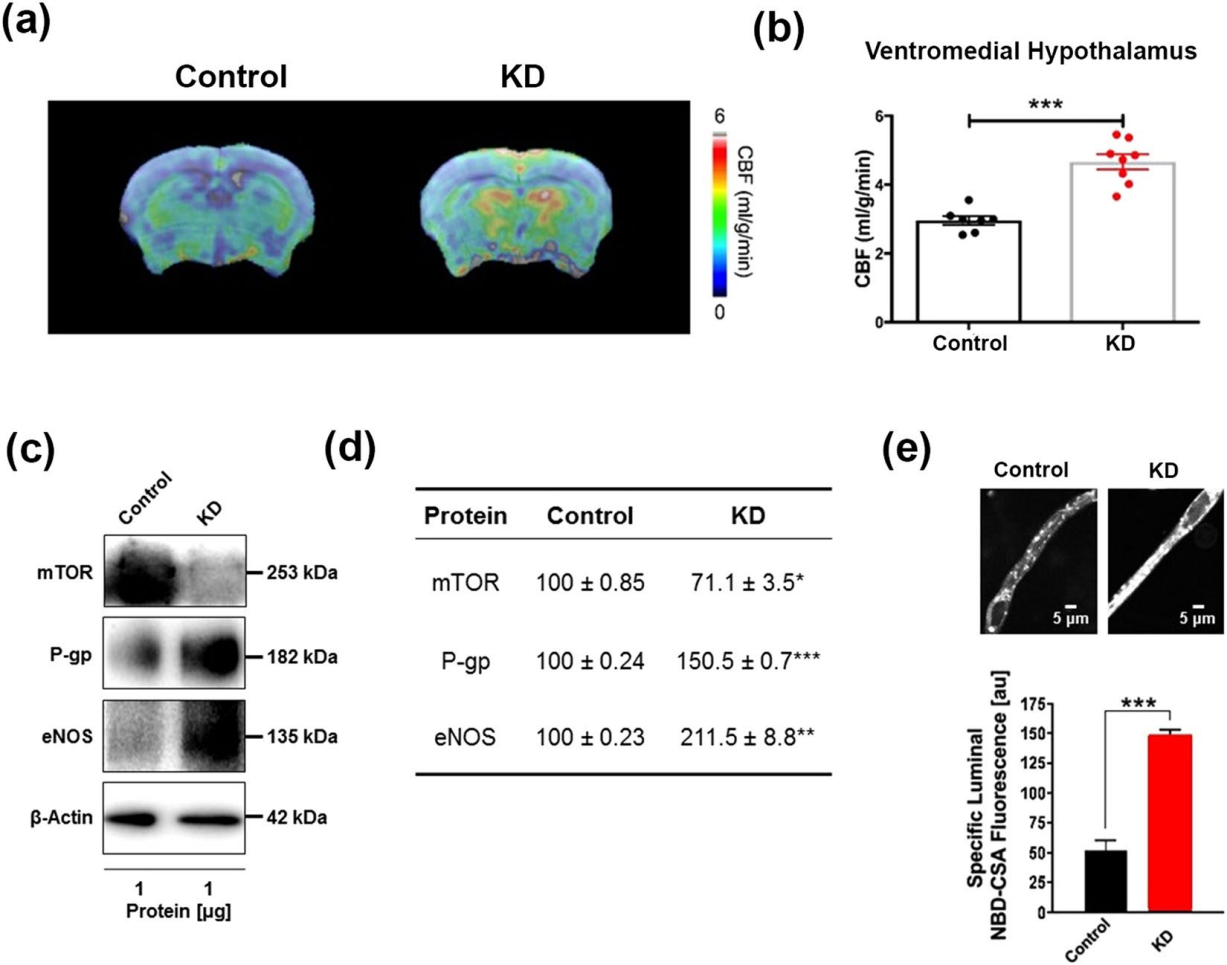
Ketogenic diet enhances neurovascular functions. (**a**) Representative cerebral blood flow (CBF) maps superimposed on structural images; color code indicates level of CBF in a linear scale. KD mice exhibited significantly higher CBF in the (**b**) ventromedial hypothalamus. Data are presented as mean ± SEM, ***p < 0.001. (**c**) Western blot (WB) images for mTOR, P-gp, and eNOS from the cortical vasculature, β-Actin was used as loading control. (**d**) The corresponding values of the levels of protein expression. WB data from KD mice were normalized to β-Actin and compared to the control mice (100%), *p < 0.05, **p < 0.01, ***p < 0.001. (**e**) Representative confocal images showing increased luminal accumulation of NBD-CSA fluorescence in brain capillaries isolated from KD mice compared to control mice, indicating higher P-gp transport activity. Corresponding quantitative fluorescence data; images are shown in arbitrary fluorescence units (scale 0–255). Data are mean ± SEM for 10 capillaries from one preparation of 10 mice per group, ***p < 0.001. mTOR: mechanistic target of Rapamycin; P-gp: P-glycoprotein; eNOS; endothelial nitric oxide synthase.

We used magnetic resonance imaging (MRI) to measure CBF and confocal microscopy to determine BBB function. Figure 1a shows representative CBF maps superimposed on structural brain images. The color code indicates the level of CBF in a linear scale. We found that KD-fed mice had significantly elevated CBF globally and regionally, particularly in ventromedial hypothalamus (VMH) (11.82%; *p* < 0.0001; Fig. 1b). We previously reported that inhibiting mechanistic target of rapamycin (mTOR) signaling increases neurovascular function by activating endothelial nitric oxide synthase (eNOS)^21,22^. In this study, we also found that mTOR protein expression was reduced (−29.9%; *p* < 0.01) while eNOS levels were increased (111.5%; *p* < 0.001) in mice fed with KD, compared to control mice (Fig. 1c). In addition, protein expression levels of P-glycoprotein (P-gp), which transports Aβ across at the BBB, were also significantly elevated in KD-fed mice (50.5%; *p* < 0.001). The Western blots are shown in Fig. 1c and the corresponding values are shown in Fig. 1d. We further used confocal microscope imaging to assess P-gp transport activity^23^. This assay measures accumulation of [N-ε(4-nitro-benzofurazan-7-yl)-DLys(8)]-cyclosporin A (NBD-CSA), a fluorescent P-gp substrate in the capillary lumen. Figure 1e (top) shows representative confocal images of capillaries incubated to steady state in a medium containing 2 µM NBD-CSA; the intensity of fluorescence in the capillary lumen reflects the amount of NBD-CSA transported by P-gp. The corresponding quantitative results are shown in Fig. 1e (bottom) – KD mice had significantly enhanced P-gp transport activity (185.38%; *p* < 0.001) in capillaries compared to capillaries isolated from control mice. Taken together, these results indicate that KD enhanced neurovascular function and increased Aβ clearance.

### Ketogenic diet alters gut microbial diversity and increases beneficial microbiota

**Figure 2:**
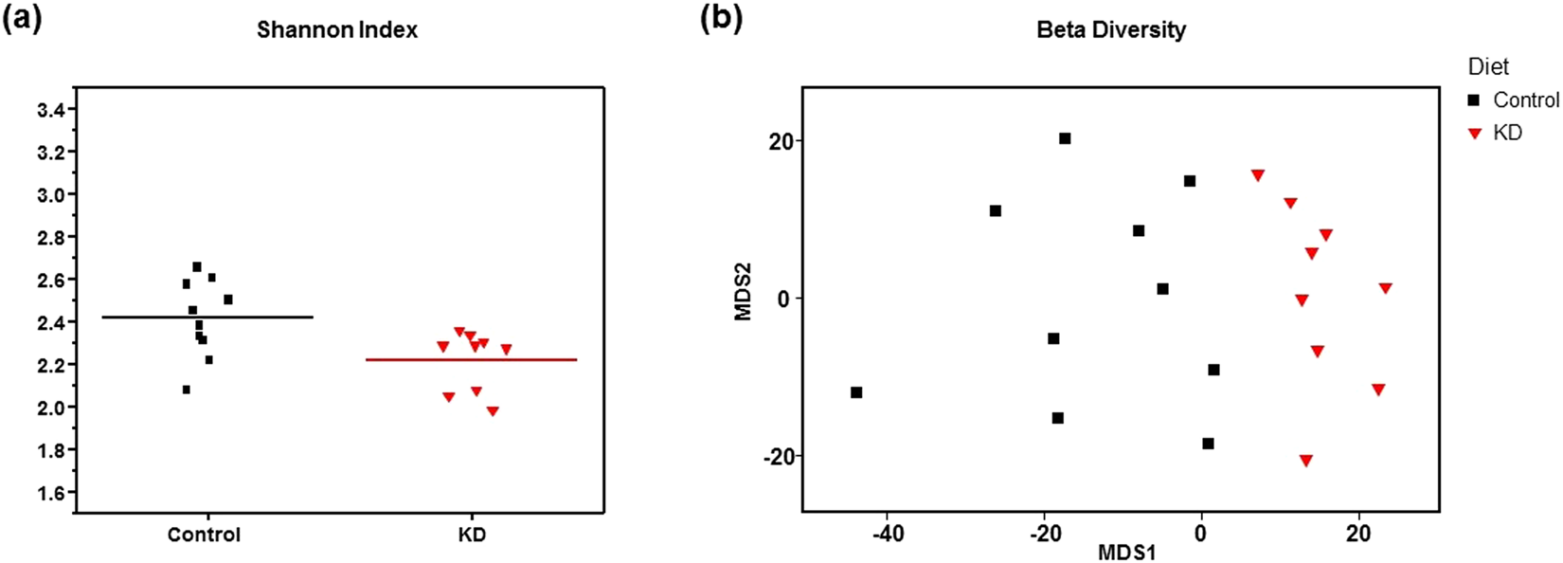
Ketogenic diet alters gut microbial diversity and increases pro-vascular microbiota. (**a**) Microbial diversity (Shannon index) was significantly higher in fecal samples from control relative to KD mice (Mann-Whitney U < 0.02). (**b**) Genus-level metric multi-dimensional scaling (mMDS) plot of 16S rRNA gene amplicon microbiome data was generated with a Bray-Curtis resemblance matrix. Fecal microbial communities of control and KD mice were significantly different in terms of individual taxa (ANOSIM R = 0.473; *p* = 0.0002), as described in the text and Table 1. All samples were standardized and square root transformed. 2D stress = 0.16.

Figure 2a shows Shannon index (H) for alpha diversity, which is measure of within-sample diversity and is a synthesis of both the richness and evenness of the microbial community in a particular sample. Shannon indices calculated based on rarefied datasets (10,000 sequences/sample) were significantly different between control and KD mice (Mann-Whitney U test; *p* = 0.018), with the fecal microbiomes of KD mice having lower diversity. Figure 2b shows a genus-level metric multi-dimensional scaling (mMDS) plot of 16s rRNA gene amplicon microbiome data generated from a Bray-Curtis resemblance matrix. The fecal microbiomes of control and KD mice were significantly different as assessed by analysis of similarity (ANOSIM; R = 0.473, *p* = 0.0002). These results indicate that KD significantly altered fecal microbial composition, and the effects included both a significant decrease in microbial diversity and a significant shift in microbial community composition.

We used a group significance test (Kruskal-Wallis test)^24^ to identify specific microbial taxa, which differed in relative abundance between fecal samples of KD and control mice. Table 1 shows the genera and species that were significantly different between control and KD mice (Kruskal-Wallis test, with Benjamini-Hochberg false discovery rate *p* < 0.05). We found that KD mice had significant increases in the relative abundance of *A. muciniphila* and *Lactobacillus* (approximately 2.5 and 3.2-fold increases, respectively). Both taxa have members that are capable of producing SCFAs^25,26^ and members of the genus *Lactobacillus* have been used elsewhere as probiotics^27^. Two low abundance genera of putative acetate producers (<0.3% relative abundance) within the order Clostridiales, *Clostridium* and *Dorea*^28^, were significantly lower in KD mice. The relative abundance of bacteria from the genera *Desulfovibrio* and *Turicibacter* was significantly and substantially lower in KD mice. *Desulfovibrio* sequences were not detected in KD mice but present at an average relative abundance of 0.53% in control animals, while *Turicibacter* relative abundance was nearly two orders of magnitude higher in the fecal samples of control mice relative to KD mice. *Desulfovibrio* are sulfate-reducing bacteria associated with inflammatory bowel disease^29^, while members of the genus *Turicibacter* were shown to increase with diet-induced obesity^30^. Collectively, KD increased the relative abundance of microbiota that are putatively able to protect neurovascular integrity, as well as reduced those which may induce inflammation.

**Table 1:** Diet changes in gut microbiome composition.

Genera with significantly different relative abundance between control and KD animals^*^	Control	KD	p-value	FDRC
**Phylum** Actinobacteria				
**Class** Coriobacteriia; **Order** Coriobacteriales; **Family** Coriobacteriaceae; **Genus** *Adlercreutzia*	28.5	60.2	0.002	0.019
**Phylum** Firmicutes				
**Class** Bacilli; **Order** Lactobacillales; **Family** Lactobacillaceae; **Genus** *Lactobacillus*	142.8	453.4	0.006	0.008
**Class** Bacilli; **Order** Turicibacterales; **Family** Turicibacteraceae; **Genus** *Turicibacter*	120.2	1.6	0.000	0.008
**Class** Clostridia; **Order** Clostridiales; **Family** Clostridiaceae; **Genus** *Clostridium*	2.1	0.2	0.002	0.019
**Class** Clostridia; **Order** Clostridiales; **Family** Lachnospiraceae; **Genus** *Dorea*	19.7	4.0	0.002	0.019
**Class** Erysipelotrichi; **Order** Erysipelotrichales; **Family** Erysipelotrichaceae; **Genus** *Clostridium*	187.0	831.2	0.003	0.020
**Phylum** Proteobacteria				
**Class** Deltaproteobacteria; **Order** Desulfovibrionales; **Family** Desulfovibrionaceae; **Genus** *Desulfovibrio*	53.4	0.0	0.001	0.015
**Phylum** Verrucomicrobia				
**Class** Verrucomicrobiae; **Order** Verrucomicrobiales; **Family** Verrucomicrobiaceae; **Genus** Akkermansia**	792.1	1943.9	0.005	0.028

*Data for ‘Control’ and ‘KD’ are presented as mean number of reads per sample, based on a rarefied sequencing depth of 10,000 sequences/sample. The p-value is derived from a Kruskal-Wallis test, and the FDRC is the Benjamini-Hochberg false-discovery rate adjusted p-value. Only those genus-level taxa with an FDRC <0.05 are shown.

**All *Akkermansia* sequences were annotated to the taxonomic level of species as *A. muciniphila*.

### Ketogenic diet modulates blood ketone and glucose, and decreases body weight

**Figure 3:**
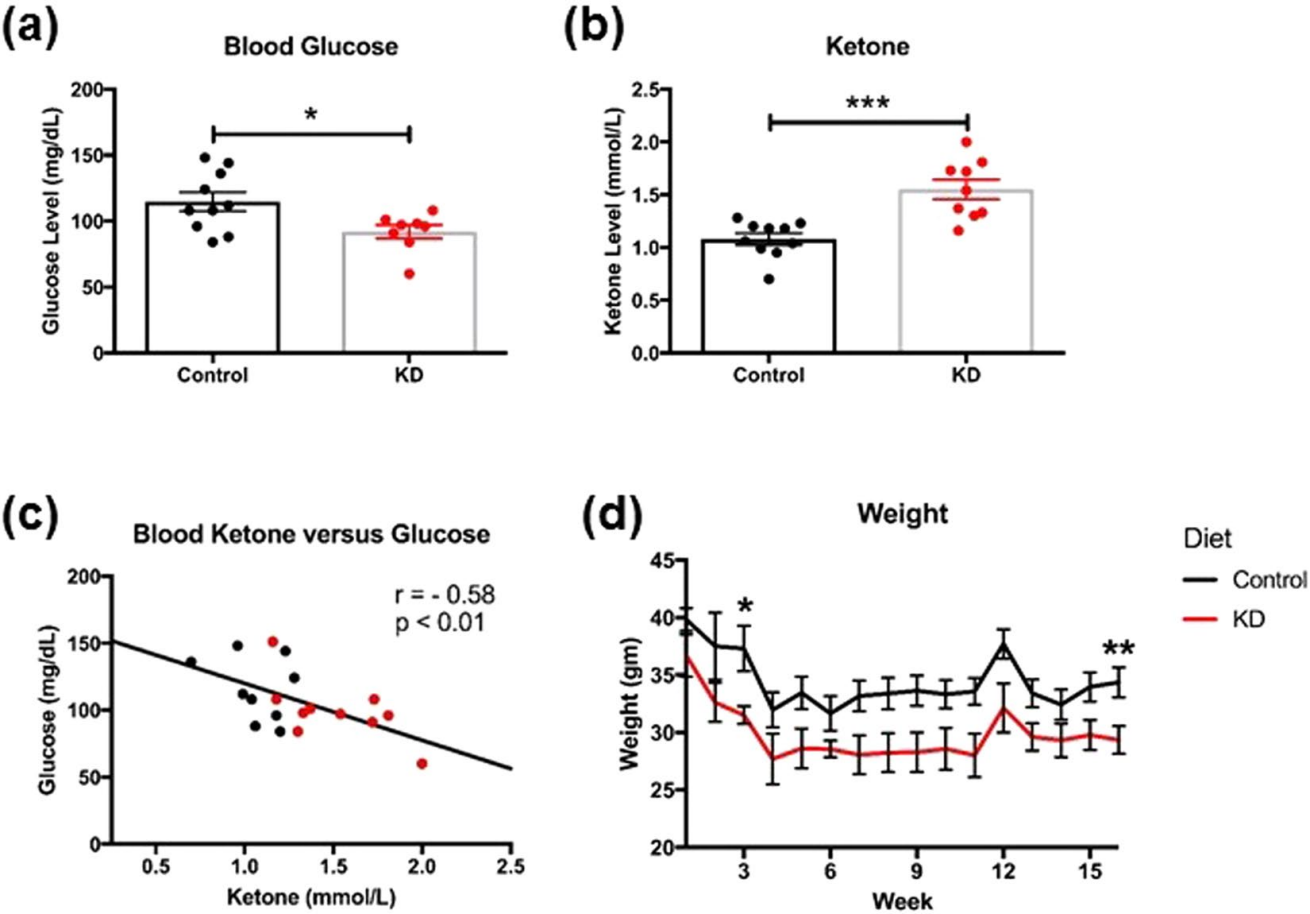
Ketogenic diet modulates blood ketone and glucose, and decreases body weight. (**a**) KD mice had significantly lower blood glucose and (**b**) significantly higher blood ketone levels than control mice. (**c**) A scatter plot of blood ketone and glucose showing an inverse linear relationship (Pearson’s r = −0.5761, *p* < 0.01) where each point represents a mouse (n = 19). (**d**) KD mice had a significant decrease in weight over the 16 weeks compared to control mice.

*A. muciniphila* can increase insulin sensitivity^31^, and exhibits a negative correlation with body-mass index^32^. Consistent with our gut microbiome findings, we observed a significant decrease in blood glucose level in the KD mice (Fig. 3a; −19.97%; *p* = 0.01). With KD feeding, mice exhibited significantly higher ketone concentration when compared to the control group (Fig. 3b; 43.48%; *p* = 0.0004). Interestingly, there was a significant inverse correlation between ketone level and blood glucose measurements (Fig. 3c; Pearson’s r = − 0.58; r^2^ = 0.33, *p* < 0.01). Another interesting aspect of the present study is that KD mice lost body weight despite having higher energy intake (7.24 kcal/gm) compared to the control mice (3.79 kcal/gm). KD mice exhibited reduced body weight by week 3 (Fig. 3d; −15.50%; *p* = 0.0096), and this trend continued for the remainder of the study. At the time of the final measurement, the KD mice maintained significantly lower weights than the control mice (−14.58%; *p* = 0.0042). Our findings are consistent with literature that KD reduces blood glucose levels^33^ and body weight^34^.

## Discussion

In the present study, we demonstrated that KD enhanced neurovascular functions and increased beneficial gut microbiota in young healthy mice. In particular, KD enhanced BBB function by increasing protein expression and transport activity of the Aβ transporter P-gp, increased CBF in VMH, and reduced mTOR with increased eNOS protein expressions. In addition, KD increased potentially beneficial gut microorganisms, including *A. muciniphila* and bacteria from the genus *Lactobacillus*. KD also reduced potentially pro-inflammatory taxa, including bacteria from the genera *Desulfovibrio* and *Turicibacter*. We also found that mice fed with KD had reduced blood glucose levels and body weight, and increased blood ketone body levels; there was an inverse correlation between blood glucose and ketone bodies levels. Collectively, KD showed multifactorial benefits for mice even under healthy conditions.

The enhancements of CBF, BBB transporter activities, and potentially increased Aβ clearance suggest that mice with KD may have lower risk to develop age-related neurodegenerative disorders, including AD. This is consistent with literature that KD reduces Aβ toxicity and restores memory in animal models that develop AD-like symptoms^35–38^. This also agrees with our previous findings showing that preserving CBF and BBB integrity is critical in preventing AD development in a mouse model with human APOE4 gene, the strongest genetic risk factor for AD^39^; and restoring CBF improves memory in a symptomatic AD mouse model^21^. Our findings are consistent with literature showing that an acute increase in ketone body levels elevated CBF in rats^37^ and humans^36^.

We further demonstrate that mTOR inhibition may play an important role in neurovascular enhancements. mTOR is a nutrient sensor and its activity can be down-regulated when glucose availability is reduced or ketogenesis is increased^40^. Similar to caloric restriction, mTOR inhibition can preserve brain metabolism with age^41^, potentially due to the shift from glucose to ketone body utilization^42^. In the present study, we found that KD mice exhibited significantly lower expression of mTOR, but significantly increased expression of eNOS. This is consistent with our previous study using rapamycin, showing that mTOR inhibition can activate eNOS, which causes the release of nitric oxide, a vessel dilator, and consequently elevates CBF^21,23^. This is also consistent with studies showing that down-regulation of mTOR is associated with elevated P-gp expression^23^. The neurovascular enhancements by mTOR inhibition were associated with preserved white matter integrity and long-term memory, and reduced anxiety in aging mice^23,43^.

Gut microbiome alterations induced by KD may also contribute to the neurovascular enhancements. We found that KD significantly increased the relative abundance of *A. muciniphila* and *Lactobacillus*, known to produce SCFAs^25–27^. SCFAs are transported by monocarboxylate transporters expressed at the BBB^44,45^. Lack of SCFAs can cause higher BBB permeability^7,8,11^. On the other hand, we found that *Desulfovibrio* was absent in KD mice. This is consistent with a prior study showing that KD significantly decreases the abundance of *Desulfovibrio* in Glucose Transporter 1 Deficiency Syndrome^29^. Members of the genus *Desulfovibrio* are capable of respiring sulfate and producing hydrogen sulfide, which is known to induce gut barrier impairment^46^. Consequently, reducing the abundance of *Desulfovibrio* may also facilitate BBB and neurovascular enhancements. Together, changes in relative abundance of *A. muciniphila*, *Lactobacillus*, and *Desulfovibrio* may contribute to protection of neurovascular functions.

We found that KD decreased overall microbial diversity. This is likely a result of reduced carbohydrate intake, which decreases the polysaccharide content that many gut bacteria derive energy from^47^. Although other studies suggested that reduced microbiome diversity may be associated with disease progression^48^, we found that levels of several beneficial microbiota were increased, even though the overall diversity was reduced by KD. In part, the decrease in diversity (Shannon index) can be attributed to the substantially higher relative abundance of *A. muciniphila* in KD mice, reaching nearly 20% of the observed microbial community. Future studies are needed to further identify the different contributions between overall diversity and underlying taxonomy for health and diseases. In addition, shotgun metagenome and metatranscriptome sequencing efforts will be required to associate specific metabolic capabilities (*e.g*., SCFAs production) with identified taxa, and to demonstrate where and when these genes are expressed *in situ* in the gastrointestinal tract.

We confirmed that KD is able to reduce body weight^34^ and lower blood glucose level^33^. This could be due to increased relative abundance of *A. muciniphila* and *Lactobacillus* spp. The relative abundance of *A. muciniphila* also increases when type 2 diabetic patients are given metformin^49^, a prescribed medication to increase glucose utilization and reduce body weight by activating AMP-activated protein kinase (AMPK) pathway^50^. Accordingly, *A. muciniphila* is associated with increased insulin sensitivity^31^ and reduced body weight^32^. Similar results could be elicited by *Lactobacillus* through SCFAs production. In line with this, *Lactobacillus* has been shown in several studies to decrease body weight and fat^51^.

Being able to maintain proper body weight and blood glucose level is crucial for reducing risk for AD, which is known as type 3 diabetes with increased glucose intolerance in the brain^52^. In fact, diabetes or obesity is highly associated with glucose intolerance, insulin insensitivity, and increased risk for AD^53^. Recent advances indicate that excessive white fat increases secretion of pro-inflammatory cytokines from adipocytes, which could consequently lead to neuroinflammation, Aβ retention, brain cell death, and dementia^54^. In a study with obese rats, KD reduced their body weight and improved their lipid profile^55^. In addition, KD decreases pro-inflammatory cytokines (e.g., TNF-α), down-regulates brain amyloid protein precursor, and improves brain oxidative stress of the obese rats. Furthermore, KD improves hippocampal glycolytic and tricarboxylic acid cycle intermediates and amino acid in a 3xTgAD mouse model^56^, suggesting that KD may also improve insulin sensitivity in the brain^57^. It indicates that KD-induced body weight loss may evoke metabolic and immune function changes that potentially lead to neuroprotective effects. This is consistent with a human study that diet-induced weight loss improves functional brain responses during an episodic memory task^58^.

Although KD mice had higher energy intake per gram of the food (due to high fat content) compared to the control mice, they still showed lower body weight. As ketone bodies could enhance fatty acid metabolism, we speculate that KD would switch from burning carbohydrate to fat and thus facilitate fat utilization more effectively in the body^59^. This is consistent with a clinical study that people with obesity lost three times more visceral adipose tissue with KD^60^.

There were several limitations in the present study. We did not measure levels of SCFAs, and tight junction protein in the intestinal barrier and BBB. Therefore, we could not identify further linkages between the gut microbiome changes and brain vascular protection. In addition, we used DNA-based amplicon profiling of the microbial community in fecal samples to determine the diversity and taxonomy of gut microbiota. We acknowledge that the various compartments of the gastrointestinal (GI) tract harbor different bacterial populations^61^; therefore, future studies will need to include different parts of the GI tracts for gut microbiome analysis, and to incorporate shotgun sequencing approaches to the community profiling to improve taxonomic resolution and to measure gene expression activity.

In conclusion, we have demonstrated that KD enhanced neurovascular functions, which might be associated with the diet-induced changes in gut microbiome. Our results indicate that KD may not only be beneficial in disease states, but also in healthy condition. These findings imply that dietary intervention started in early stages may evoke neuroprotective effects via neurovascular and gut microbiome changes. Future studies will be needed to further identify the mechanism linking brain and gut interactions for KD-induced neuroprotective effects in both healthy and disease states. Understanding nutritional effects on central and enteric nervous systems, and their interactions, has profound implications for neuroprotection in humans.

## Materials and Methods

### Animals and Diet

We used C57BL/6 male mice (12–14 weeks of age) obtained from the National Institutes of Health. We determined the sample size with the power that could perform the comparison at a 0.05 level of significance, with a 90% chance of detecting a true difference of all the measurements between the two groups; N = 9–10 per group were used in the study. After arriving at our facilities, each mouse was given its own cage housed in a specific pathogen-free facility to avoid microbiome transfer^62^. The control regimen (type F1515) consisted of 3.79 kcal/gm pellets composed of 65.2% carbohydrates, 18.1% protein, 5.1% fat, 4.8% fiber, 2.9% ash, and less than 10% moisture. The KD regimen (type F3666), in the form of a 7.24 kcal/gm paste, consisted of 75.1% fat (composed of saturated, monounsaturated, and polyunsaturated fatty acids), 8.6% protein, 4.8% fiber, 3.2% carbohydrates, 3.0% ash, and less than 10% moisture. Both diets were obtained from Bio-Serv. All mice were fed *ad libitum* for 16 weeks, and body weight was measured once a week. The amount of remaining diet was weighted each week to determine the food intake of the mice. We did not find a significant difference in food intake between the control (46 ± 3 g/mouse/week) and KD mice (47 ± 2 g/mouse/week; *p* > 0.5). All experimental procedures were approved by the Institutional Animal Care and Use Committee (IACUC) at the University of Kentucky (UK) and in compliance with the ARRIVE guidelines^63^.

### Cerebral Blood Flow Measurement

We measured CBF using MRI-based arterial spin labeling techniques. Details have been described in a previous study^23^. Briefly, MRI experiments were performed on a 7T MR scanner (Clinscan, Bruker BioSpin, Germany) at the Magnetic Resonance Imaging & Spectroscopy Center of the University of Kentucky. Mice were anesthetized with 4.0% isoflurane for induction and then maintained in a 1.2% isoflurane and air mixture using a nose cone. Heart rate (90–110 bpm), respiration rate (50–80 breaths/min), and rectal temperature (37 ± 1 °C) was continuously monitored and maintained. A water bath with circulating water at 45–50 °C was used to maintain the body temperature. A whole-body volume coil was used for transmission and a mouse brain surface coil was placed on top of the head for receiving. We measured CBF using MRI-based pseudo-continuous arterial spin labeling (pCASL) techniques^23^. Paired control and label images were acquired in an interleaved fashion with a train of Hanning window-shaped radiofrequency pulses of duration/spacing = 200/200 μs, flip angle = 25° and slice-selective gradient = 9 mT/m, and a labeling duration = 2100 ms. The images were acquired by 2D multi-slice spin-echo echo planner imaging with FOV = 18 × 18 mm^2^, matrix = 128 × 128, slice thickness = 1 mm, 10 slices, TR = 4,000 ms, TE = 35 ms, and 120 repetitions. pCASL images were analyzed with in-house written codes in MATLAB (MathWorks, Natick, MA) to obtain quantitative CBF (with units of mL/g per minute). Brain structural T_2_-weighted images were acquired with field of view (FOV) = 18 × 18 mm^2^, matrix = 256 × 256; slice thickness = 1 mm, 10 slices, repetition time (TR) = 1500 ms, and echo time (TE) = 35 ms. The CBF images were then superimposed to the corresponding structural images using Multi-Image Analysis GUI (Mango) software (http://rii.uthscsa.edu/mango/).

### Gut Microbiome Analyses

#### Fecal DNA Amplification

The experimental protocol has been described in a previous study^64^. Fecal samples were collected from control and KD mice and frozen at −80 °C until further use. The PowerSoil DNA Isolation Kit (MO BIO Laboratories, Inc.) was used for fecal DNA extraction, according to the manufacturer’s protocol. Genomic DNA was PCR amplified with primers 515 F modified and 926R^65^ targeting the V4-V5 regions of microbial 16S rRNA genes using a two-stage “targeted amplicon sequencing (TAS)” protocol^66^. The primers contained 5′ common sequence tags (known as common sequence 1 and 2, CS1 and CS2) as described previously^67^. First stage amplifications were performed with the following thermocycling conditions: 95 °C for 3 mins, 28 cycles of 95 °C for 45 sec, 50 °C for 45 sec, 72 °C for 1:30 minutes and final elongation at 72 °C for 10 minutes. The PCR master mix was made in a total volume of 25 µl of reaction mixture containing 4 µl (100 ng) of DNA template, 0.5 µl (20 µM) of each forward and reverse primers, 12.5 µl of PCR ready-to-use mixture (MyTaq HS Mix 2x, Bioline, London, UK) and 7.5 µl of distilled water.

Subsequently, a second PCR amplification was performed in 10 microliter reactions in 96-well plates. A mastermix for the entire plate was made using the MyTaq HS 2X mastermix. Each well received a separate primer pair with a unique 10-base barcode, obtained from the Access Array Barcode Library for Illumina (Fluidigm, South San Francisco, CA; Item# 100-4876). These Access Array primers contained the CS1 and CS2 linkers at the 3′ ends of the oligonucleotides. Cycling conditions were as follows: 95 °C for 5 minutes, followed by 8 cycles of 95 °C for 30”, 60 °C for 30” and 72 °C for 30”. A final, 7-minute elongation step was performed at 72 °C. PCR products were purified using SequalPrep plates (Life Technologies) according to the manufacturer’s instructions. Subsequently, these PCR products were quantified using a Quant-iT PicoGreen dsDNA Assay Kit (Thermo Fisher), implemented on a Genios Pro Fluorescence microplate reader (Tecan). PCR products were then pooled using PicoGreen quantification results, using an epMotion5075 liquid handling workstation (Eppendorf).

The pooled libraries, with a 15% phiX spike-in, were loaded on to a MiSeq v3 flow cell, and sequenced using an Illumina MiSeq sequencer, with paired-end 300 base reads. Fluidigm sequencing primers, targeting the CS1 and CS2 linker regions, were used to initiate sequencing. De-multiplexing of reads was performed on instrument. Second stage PCR amplification and library pooling was performed at the DNA Services (DNAS) facility, Research Resources Center (RRC), University of Illinois at Chicago (UIC). Sequencing was performed at the W.M. Keck Center for Comparative and Functional Genomics at the University of Illinois at Urbana-Champaign (UIUC).

#### Microbial Analysis

Forward and reverse reads were merged using PEAR^68^. Primer sequences were identified using Smith-Watermann alignment and trimmed from the sequence. Reads that lacked either primer sequence were discarded. Sequences were then trimmed based on quality scores using a modified Mott algorithm with PHRED quality threshold of p = 0.01, and sequences shorter than 300 bases after trimming were discarded. QIIME v1.8 was used to generate OTU tables and taxonomic summaries^24^. Briefly, the resulting sequence files were merged with sample information. OTU clusters were generated in a *de novo* manner using the UCLUST algorithm with a 97% similarity threshold^69^. Chimeric sequences were identified using the USEARCH61 algorithm with the GreenGenes 13_8 reference sequences^70^. Taxonomic annotations for each OTU were using the UCLUST algorithm and GreenGenes 13_8 reference with a minimum similarity threshold of 90%^69,70^. Taxonomic and OTU abundance data were merged into a single OTU table and summaries of absolute abundances of taxa were generated for all phyla, classes, orders, families, genera, and species present in the dataset. The taxonomic summary tables were then rarefied to a depth of 10,000 counts per sample.

Shannon and Bray-Curtis indices were calculated with default parameters in software package Primer7^71^. The rarefied species data, taxonomic level 6, were used to calculate both indices. Significant difference among tested groups was determined using the Kruskal-Wallis one-way analysis of variance. The group significance tests were performed on the rarefied species data using the group_significance.py script within the QIIME v1.8 package. The gene amplicon sequence data generated as part of this study have been submitted to the NCBI BioProject database (PRJNA401034).

### P-glycoprotein (P-gp) Transport Determination and Western Blotting

#### Capillary isolation

Details of the experiments have been described in previous studies^23,64^. Brain capillaries were isolated from mice according to a previously described protocol^23^. Briefly, mice were euthanized by CO_2_ inhalation and decapitated; brains were immediately harvested and collected in ice-cold DPBS buffer supplemented with 5 mM D-glucose and 1 mM Na-pyruvate, pH 7.4. Brains were dissected by removing meninges, choroid plexus and white matter, and homogenized in DPBS. The brain homogenate was mixed with Ficoll^®^ and centrifuged at 5,800 g for 15 min at 4 °C. The capillary pellet was resuspended in 1% BSA buffer and first passed through a 300 µm nylon mesh followed by filtration through a 27 µm nylon mesh. Capillaries retained by the 27 µm nylon mesh were collected and washed with DPBS buffer, and used for experiments.

#### P-glycoprotein transport activity

Isolated brain capillaries were incubated for 1 h at room temperature with 2 μM NBD-CSA (custom-synthesized by R. Wenger, Basel, Switzerland) in DPBS buffer. Per treatment group, 10 capillary images were acquired by confocal microscopy (Leica TSP SP5 Confocal Microscope with Environmental Chamber, 63 × D-Water UV objective, numerical aperture 1.2, 488-nm line of an argon laser, Leica Microsystems). Confocal images were analyzed by quantitating luminal NBD-CSA fluorescence with Image J software (v.1.45 s; Wayne Rasband, NIH). Specific, luminal NBD-CSA fluorescence was taken as the difference between total luminal fluorescence and fluorescence in the presence of the P-glycoprotein specific inhibitor PSC833 (5 μM, Novartis, Basel, Switzerland).

#### Western blotting and quantification

To determine protein expression, isolated brain capillaries were homogenized in tissue lysis buffer containing protease inhibitor cocktail. Homogenized brain capillary samples were centrifuged at 10,000 g for 15 min at 4 °C, followed by centrifugation of the denucleated supernatants at 100,000 g for 90 min at 4 °C. Pellets (crude brain capillary plasma membranes) were resuspended and protein concentrations were determined using the Bradford protein assay. Western blots were performed using the NuPage™ electrophoresis and blotting system from Invitrogen (Carlsbad, CA, USA). Blotting membranes were incubated overnight with antibody to P-gp (C219; MA1-26528, ThermoFisher, 1 μg/ml), mTOR (ab134903, Abcam, 1 μg/ml), GLUT1 (ab652, Abcam, 1 μg/ml), and β-actin (ab8226 from Abcam, 1:1000, 1 μg/ml). Proteins were detected using SuperSignal^®^ West Pico Chemoluminescent substrate (Pierce, Rockford, IL, USA) and protein bands were visualized with a BioRad Gel Doc™ XRS imaging system. Image Lab 5.0 software from Bio-Rad Laboratories was used for densitometric analyses of band intensities and digital molecular weight analyses; the molecular weight marker was RPN800E (GE Healthcare, Chalfont St. Giles, Buckinghamshire, UK). Linear adjustments of contrast and brightness were applied to entire Western blot images. None of the Western blots shown were modified by nonlinear adjustments.

### Blood Glucose and Ketone Bodies Measurements

The procedure has been described in a previous study^43^. When the mice were sacrificed, blood samples were collected in 500 μl lithium heparin 12.5 IU Terumo Capiject Capillary blood collection tubes (Vacutainer K2 EDTA) to avoid blood coagulation. A total of 1–2 μl of blood sample were used to measure blood glucose level using a blood glucose meter and a test strip (Clarity Plus, Boca Raton, FL, USA). Another 10 μl of blood sample was used for ketone bodies level measurement using a STAT-Site M (β-Hydroxybutyrate) meter and a test strip (Standbio Ketosite STAT-Site M-β HB, Boerne, TX, USA).

### Statistics

Statistical analyses were performed using GraphPad Prism 7 (GraphPad, San Diego, CA, USA). One-tailed Student’s *t*-test was performed for determination of differences between the two groups. Values of *p* < 0.05 were considered statistically significant.
